# “Work Like a Chinese”: Aspirations, Patterns of Work, and Working Conditions of the Chinese Immigrant Community in Southern Spain

**DOI:** 10.3390/ijerph17197063

**Published:** 2020-09-27

**Authors:** Bárbara Badanta, María González-Cano Caballero, Elena Fernández-García, Rocío de Diego-Cordero, Giancarlo Lucchetti, Rafael-Jesús Fernández-Castillo, Sergio Barrientos-Trigo

**Affiliations:** 1 Research Group PAIDI-CTS 1050 Complex Care, Chronicity and Health Outcomes, University of Seville, 41009 Seville, Spain; bbadanta@us.es (B.B.); efernandez23@us.es (E.F.-G.); sbarrientos@us.es (S.B.-T.); 2 Faculty of Nursing, Physiotherapy and Podiatry, Department of Nursing, University of Seville, 41009 Seville, Spain; mgonzalez79@us.es (M.G.-C.C.); rfernandezc@us.es (R.-J.F.-C.); 3 Research Group CTS 969 Innovation in HealthCare and Social Determinants of Health, University of Seville, 41009 Seville, Spain; 4 Department of Medicine, School of Medicine, Federal University of Juiz de Fora, Juiz de Fora 36036-900, Brazil; g.lucchetti@yahoo.com.br

**Keywords:** working conditions, occupational health, Chinese, emigrants or immigrants

## Abstract

Expanding businesses was the main reason for the immigration of Chinese people in Spain, which consists the fifth largest nationality of immigrants in this country. Nevertheless, few studies have been carried out to understand the working conditions of this population. Using an ethnographic design, this study examined the work patterns and working conditions among Chinese immigrants living in southern Spain and how these factors affected their health. Observing participants, field notes, and semi-structured interviews with question script were conducted with 133 Chinese immigrants. Five main themes were defined: “Economic improvement as a migratory reason”, “Conception to Work”, “Labor Sector”, “Work conditions”, and “Occupational health”. Our results showed that Chinese immigrants worked in the provision of services, with long working hours and little rest. Although they had low rates of unemployment, the working conditions had an important impact on their dietary patterns and their family life. Ergonomic and psychosocial risks also explained high rates of musculoskeletal problems and stress. In conclusion, Chinese immigrants living in southern Spain work actively in the service sector of the economy, but with many work hours. These characteristics seem to impact their health at a physical, psychological, and social level.

## 1. Introduction

The last global economic crisis that began in 2007, affected most economic sectors in both developed and developing countries [1]. The lack of job opportunities and the associated feeling of insecurity have led to an increase in international migration in recent years to countries with higher economic levels [2]. According to Eurostat data, 3.9 million people immigrated to one of the member states of the EU-27 in 2018, in which Spain ranked second (643,000) in reception of immigrants in Europe [3].

Although immigrants in the host country comprise a substantial proportion of the low-paid workforce [4], the growing crises in sectors such as manufacturing, construction, and services, caused the departure of foreign population living in Spain [1]. While the number of people of Romanian or Moroccan nationality decreased in the first half of 2017, other nationalities—such as Italians and Chinese—grew in absolute terms [5].

The entry of Spain into the European Economic Community in 1986 made this country a good destination for the expansion of import and clothing businesses by the Chinese population [6]. Upon arrival in Spain, the opening of Chinese restaurants led to the location of this population in the most important cities of the country, as well as in the coastal areas. In an attempt to guarantee a strategic location, to minimize competition, and to search for new market opportunities, Chinese immigrants began the so-called “first expansion into the interior” in the 1990s. In this way, they migrated to other large cities, which explains the presence of Chinese populations in Madrid, Cataluña, and Canary Islands, with strong growth in the 1980s, and in the Valencian Community and Andalusia approximately five years later [7].

Actually, Spain holds the fourth place in the European ranking in terms of volume of citizens from China. From 2005 to the present, this group has been the most numerous among the Asian group [2], constituting the fifth largest nationality of immigrants in Spain (4.46%). In addition, Chinese immigrants aged 16–64 years constitute 72.7% of Asians at the moment in Spain [8]. In 2019, the Chinese population in Andalusia constituted 9% of the total Chinese immigrants in Spain and it was the third largest foreign nationality (22,280 inhabitants) behind the Moroccan (145,076 inhabitants) and Romanian (79,264 inhabitants). It should be noted that the Chinese presence in Spain has maintained steady growth since 2006, despite declining immigrant populations of other nationalities due to the economy crisis that Spain suffered since 2008 [9].

Although some studies indicate that the effect of the Chinese population in Spain have been positive for economic investment, the generation of employment and its social and productive integration [2], there are few studies that analyze the working conditions of the Chinese population and their relationship and impact on health in this context.

Most of the studies were carried out in the United States, such as the case of the following three studies. A previous study has found that Chinese immigrants who worked for long hours were less legalized and were less likely to see a doctor when they got sick [10]. Likewise, other study found that working hours, interpersonal relationships at work, training, and economy had an important influence on participants’ well-being [11]. Finally, another study identified that career barriers among Chinese immigrants are related to ethnic stereotypes to language, lack of social support, and workplace discrimination [12].

The results are even scarcer while searching for studies in Europe. A previous Spanish study found that the work schedules of Chinese immigrants influenced their eating habits [13]. In Italy, a study investigated Chinese women and found that working less due to chronic pain is not considered an option to them, since work is extremely important in Chinese culture [14]. As noted above, there is a large gap for studies in Europe, which may be justified by the difficult access to this population and by the fact that this migration has become more important in the last two decades. Thus, it is important to have more studies investigating the job conditions and the health of this population in a deeper way in order to understand their needs.

In the Chinese population, although the idea of returning to the homeland after retirement is present, sometimes it is not fulfilled due to personal and family circumstances. Therefore, health problems may arise from remaining in Spain and exposure to certain occupational risks. Since job security is a global concern, it is necessary to know the working conditions of this group to promote a safe and healthy work environment [15]. Therefore, the present study aims to investigate the patterns of work and working conditions among Chinese immigrants living in southern Spain, and understand how these factors may affect this population’s health.

## 2. Materials and Methods

### 2.1. Design

An ethnographic qualitative design was adopted [16], as this allows us to explore a particular topic in a specific context, and perform in subcultural groups rather than in entire societies. Data collection consisted of participant observation, field notes, and semi-structured interviews with Chinese immigrants. All data collection was carried out by the main researcher (B.B.) over six months in 2016/2017.

### 2.2. Setting and Sample

The study was conducted in Andalusia, the southernmost region of Spain and Europe, where according to National Statistics Institute, 25% of the Chinese population of the region is concentrated [5].

The focus was on Chinese immigrants’ shared behaviors and experiences. Participants were recruited through Chinese businesses (e.g., bazaars, restaurants, grocery stores, fashion stores, technology stores, and wholesale businesses) and community institutions (e.g., educational institutions, Asian cultural centers, and health services) and, to increase the number of participants, a ‘snowball sampling’ procedure was used.

Participants were included if they were adult immigrants of Chinese origin; emigrated to Spain; were between 16 and 65 years old (working age group) and were able to communicate (in fluent Mandarin Chinese, Spanish, or English). A total of 252 businesses and institutions were visited. The intention was to increase the relevance of our findings within the Andalusia area and their generalizability to other Spanish areas. Based on these inclusion criteria, our study sample included 133 participants.

### 2.3. Procedure

In line with an ethnographic approach, observations were performed in participants’ workplaces, while the Chinese worked and attended to customers. Observational data was recorded as hand-written field notes, focusing on workplaces, work conditions, and the ways workers related to each other. Field notes included information about witnessed events, verbatim verbal exchanges, and the researcher’s personal interpretations of events. This field work was obtained in all research encounters aiming to observe the establishments, the relationship between employees and employers and the activities carried out, with a specific focus on the Chinese workers and their behavior. The field work occurred even when no interview was conducted, since the researcher was still observing individuals not interviewed and all other aspects of the environment. This observation allowed a more complex analysis of the phenomenon, not relying solely on interviews. Interviews were carried out face-to-face and lasted 30–50 min. They were audiotaped and transcribed verbatim by the main researcher (B.B.). Data collection continued until saturation criteria.

### 2.4. Instruments

The interview script was based on the *National Health Survey of Spain (NHSS 2017)* [17], a nation-wide study collecting information on the population living in Spain, which included sociodemographic data, health status and associated factors. A group of experts in transcultural health (*n* = 3) carried out an analysis of the content of the script to assess the adequacy of it. All interviews were conducted using open-ended questions starting with: “What is your work experience since you are in Spain?” and followed by other questions, such as: “How would Chinese immigrants define the concept of ‘work’ in the Chinese context?”; “What is the importance of work in the life of the Chinese immigrant population?”; “What aspects of work do Chinese people consider most important?”; “What are the working conditions and the type of employment of the Chinese immigrant population?”; “What labor risks are Chinese immigrants most exposed to?”; “How is the relationship between work and health?”; and “Does the work affect their health in a positive or negative way?”

At the same time, the interview with Chinese immigrants included a part of Background, consisting of: Demographic information (sex, age, level of education, and length of residence in Spain), Labor information such as occupation (current and last labor or profession), incomes, labor/work regime (self-employed, employed, or unemployed), and type of working day (working hours), and Health status such as satisfaction and work stress, main health problems, limitations of the main activities due to health problems and accidents. We use more closed questions in the background, allowing an extraction of data that were exposed quantitatively.

### 2.5. Data Analysis

The qualitative analysis was carried out following the steps proposed by Braun et al. [18]: (a) familiarization with the data; (b) generation of categories; (c) search, review, and definition of themes; and (d) final report. The data obtained was captured through audio recording, the use of a field diary, and the description of the settings. Since some statements were recorded in Chinese, the following translation process was carried out: a Chinese–Spanish translation by Chinese native (*n* = 2), and a Spanish–Chinese back-translation by an experienced translation company.

Transcription, literal reading, and thematic categorization were performed, and the NUDIST Nvivo (version 11) software was used. Data analyses started with individual repeated readings. The researchers (B.B., S.B., E.F.-G.) read all field notes and interview transcriptions several times. The other authors (G.L., R.D., R.-J.F.-C., and M.G.-C.) read samples of the field notes and interviews to obtain understanding. The analysis continued by organizing descriptive labels, focusing on emerging concepts and similarities/differences in participants’ behaviors and statements. The coded data from each participant were examined and compared with the data from all the other participants to develop categories of meanings. Five main themes were defined to reflect all of the categories: “Economic improvement as a migratory reason”, “Conception of Work”, “Labor Sector”, “Work conditions”, and “Occupational health” A final report was prepared with the statements of the interviewees “I-questionnaire number, sex, age”. This research followed the criteria of The Consolidated Criteria for Reporting Qualitative Studies (COREQ) (Supplementary Material Table S2).

Descriptive analysis of the background information was carried out using absolute and relative frequencies, as well as mean, median, and standard deviation.

### 2.6. Ethical Considerations

The study was approved by the Andalusian Research Ethics Committee, Spain (Code: 0873-N-16). All participants received written and oral information about the study, including the right to withdraw and the guarantee of anonymity. Informed consent was obtained from all individual participants included in the study.

## 3. Results

The usual scene when arriving at the business was to see several workers, sometimes accompanied by relatives who, without working officially, helped them in the stores. The presence of babies or school-age children who came to the store after school and who collaborated with their parents in the attention and communication with customers was also common. The main researcher was able to observe that Chinese immigrants working has a great influence in their family routine. While not attending customers, they phoned their relatives, prepared food for the children, helped children with homework, watched Chinese TV series, played games using their smartphones, and rested their legs.

In relation to work activity, the women were taking payment for the customers, while the men were handling merchandise and in the warehouses. Occasionally, employees refused to participate when the boss was in the business and they feared a quarrel. On the other hand, people who had been living in Spain for more time, worked with family members in a more relaxed environment and with a self-employed regime, showed less reluctance to participate.

In general, a young group is detected (*M* = 30.7 years), with an average length of residence in Spain of 11.3 years. The majority of Chinese immigrants come from rural areas of China (Zhejiang) (78.3%), and they are Han people, the dominant ethnic group of China. Recognizing limited possibilities of work in their place of origin, and having a medium-low educational level (71%), led them to emigrate to change their family situation: (I-62, man, 32 years) “Most of the Chinese in Spain have a very low cultural level, so all they want to do here is work”. More details about the participants are shown in Table 1.

The five main themes and the most important categories and supporting statements are shown in Table S1, as a Supplementary Material. In addition, Table 2 presents a summary for the quantitative analysis of the interview data collected.

### 3.1. Economic Improvement as a Migratory Reason

The reason for migrating is related to economic progress and therefore, they link it with work activity: (I-40, man, 53 years) “To earn money and get rich is the goal of a high percentage of the Chinese”. Normally the family decides that the young man will migrate first. The main support for the Chinese population is to be financed by family and friends in their project, both from the country of origin and destination. It is common in this group to lend money without any claim of benefit, being sure that it will be returned when the immigrant manages to earn enough money with his own business: (I-68, man, 30 years) “We lend money to each other [to migrate or create your own business], we do not ask for mortgages if we can avoid it, we do not ask for loans (...) then we are repaid without any interest”. The expectations of the person who migrates is to be able to gather the rest of his family a few years later and continue the family business.

Although the main research has observed the happiness of the Chinese immigrants when the family was reunited in Spain, in some occasions, this could also lead to stress. As an example, in one of the interviews, the five-year-old daughter of a participant had just arrived in Spain and, this situation has generated both happiness and anguish. The anguish was created by the fact that the father needed to promptly prepare all the necessary documentation for the girl to go to school, but he did not know how to do it and he did not have sufficient time to do that. The same problem with the acquisition of documentation has been also observed in another family with a two-month-old girl.

Although the initial wishes are to save money to return to China and live a comfortable retirement, the outcome usually varies due to differences in acculturation processes of family members, those born in China and those born in Spain (ethnic Chinese): (I-127, woman, 44 years) “The idea is to work here and return to China (…) with a certain economic tranquility. But truly from the experience of my parents and their environment, in the end nobody has left”.

### 3.2. Conception of Work

The conceptualization of work has a positive connotation, appearing a negative one when the person does not work: (I-81, woman, 26 years) “If you don’t work, you are useless”. Employment is the main reason why they migrate to Spain and they take it as a way of life. Chinese immigrants conceive it as a way to make money to improve their quality of life, so they want to be always working. An example of this assumption is the fact that many businesses in Spain close for lunch. However, the Chinese businesses stay open all day as observed by the main researcher in her fieldwork. In fact, dedicating as much time as possible to work is a characteristic learned in China: (I-88, man, 35 years) “Everyone says to work like a Chinese (...). People are already used to working hard (...) and not like in Spain, if you do not work the government helps you, but in China there is such a large population and if you do not work, nobody will help you”. Furthermore, working becomes the only way to live in the place of destination due to cultural differences with the host society: (I-35, woman, 19 years) “If I was in China today, I would leave before work for the Chinese New Year party… but not here in Spain”.

### 3.3. Labor Sector

Among all businesses randomly visited by the main researcher, a greater number of shops and bazaars were identified. Through interviews, commerce was quantified as the main sector in which participants were working, specifically bazaars and food stores (36.8%), clothing stores (16%), and wholesale stores (12%). This was also supported when the interviewees spoke about their last job, so changes in trends were detected. It was discovered that 56.6% worked in the commerce area (wholesale or retail trades), and that eating establishments also predominated (28.3%). Regarding to differences between previous and current jobs, the eating establishments have now decreased to be occupied by 8% of the interviewed. In the commercial sector, there has been an increase to 85%.

In fact, most of the businesses that they occupy are similar since they do not require a very high level of management: (I-8, man, 41 years) “As the majority who came have a low educational level, they set up the easiest: import, bazaar, shoes (...) greengrocers, bars ... The only thing they would not set up would be because of their education limits (...). As they think that it escapes their control, they do not want to get involved”.

However, the change from one sector to another has fluctuated according to market saturation: (I-30; man, 41 years) “A few years ago the eating establishments predominated. This tendency has been decreasing (...). Here in Spain most are wholesalers or bazaars”. Comparing the appearance of Chinese businesses from a few years ago in relation to the present, an aesthetic improvement has been observed, and the interior appearance is very similar to other stores that predominate in Spain. The Chinese immigrants have made a real effort to attract customers and it is becoming difficult to identify the Chinese shops. This occurs with fashion stores, where the organization of the shelves and clothing, the prices and even the quality of the products follow Western standards and are comparable to other Spanish businesses.

In addition, the trend of restaurants has also diminished by China’s commercial explosion as the world’s largest factory, linked to wholesalers. However, Chinese immigrants are also adapting restaurants to the needs of the Spanish population. It was found that most of the restaurants had stickers next to the entrance door that referred to the possibility of delivering food at home (e.g., Just Eat), something that could save the business, keeping prices low and offering a different cuisine.

Finally, Chinese students and entrepreneurs are also working in the world of large companies and imports, as well as professionals with university studies. 

### 3.4. Work Conditions

Most of the interviewees receive income from work (96.2%), and they have the assurance of having regular work. Due to the ease of having help from other compatriots and relatives, the rate of unemployment among Chinese immigrants is very low, since practically everyone starts working from the day they arrive: (I-119, woman, 20 years) “When I came I could work in a restaurant with my father and my uncle, but I wanted to go to the city, so in China I already organized that I would work and live with my boss’s family”. Employability is similar in men and women, highlighting the important role of Chinese women as entrepreneurs, who increase their work power when speaking the Spanish language better than their husbands.

The main idea of any Chinese immigrant is to work first in the store or business of a friend or relative as an employee (24%) and save enough money to later open their own business (58%): (I-133, woman, 41 years) “Those who work for others do so temporarily until they can work on their own, which is our ultimate goal”.

In relation to workday, almost all respondents report having split work day, predominantly the days of more than 10 h continuous (I-72, woman, 43 years) “many hours of daily work without rest or closure during the half day and including full weekends”. This would explain why some people bring prepared food from home and the existence of kitchens in the back of the businesses observed by the main researcher, where Chinese immigrants eat quickly and continue the workday.

Finally, the long working hours make Chinese immigrants try to live as close as possible to work. Only few people report that some Chinese stay to sleep in their own businesses and the weekend is when they go home. On the other hand, this explains the presence of children in workplaces, sometimes for longer hours than in their own home: (I-130 man, 53 years) “My father had a restaurant, so the restaurant’s kitchen was the living room of my house. We only went to sleep at my house (...). If you go to a 1-euro store [The business idea of the “Everything for €1” store focuses on the retail market. In this type of business there is an extensive range of products, which do not have to be all under the same price (€1), but they need to respect that price range. Therefore, the sale of these products is more conditioned by the price than by the type of product. Another essential feature is that they are articles of daily use, that is, products that people need every day. The type of customer of these stores ranges from consumers looking for a product to solve an emergency or immediate need, to customers who are looking to save money on their purchases], if they have children, you often find it there. That greatly affects family organization”.

### 3.5. Occupational Health

Due to the time that the Chinese population spends working, health problems arise in the statements. While some health risks are specific: (I-38, man, 41 years) “Those who work in restaurants... being all day or many hours in the kitchen with poor ventilation can cause some risks for the respiratory tract”, most mentioned musculoskeletal problems (28.6%). Although they point out that working conditions are equal for women and men, it is observed during visits, that the heaviest physical work is mainly done by men. Other problems are those related to the lifestyles adopted because of the work conditions and stress, such as sedentary lifestyle and lack of physical activity, gastric problems, and increase in blood pressure. It was also common to observe that they did not eat in appropriate spaces (e.g., dining rooms), they ate very quickly and in little quantity in order to return to their tasks as soon as possible. However, 96.2% stated that illness or health problems in the last twelve months did not limited their usual work activity: (I-100, woman, 25 years) “Have you ever heard that the Chinese don’t get sick? If a Chinese goes to the hospital, it means that that person must be very sick. In China, since we pay for doctors, we don’t visit them that often”. Definitely, only in serious situations Chinese immigrants clearly prioritize health over work. (I-6, man, 39 years; C-131, woman, 27 years) “If you go to the doctor an appointment at 11 o’clock, you will stay there at least until noon. I’m working, so I can’t afford to waste that much time, so I prefer not to go”.

The accidents were used as another risk indicator for occupational health. The general perception of an accident at work was equated with serious situations that required complex health care and even hospitalization; hence, 88.7% of people of Chinese origin stated that they had not suffered an accident in the last 12 months. Among the people who had an accident, 53.3% suffered contusions, bruises, or sprains, while 20% did not have any damage. Some small wounds and cuts, or musculoskeletal discomfort caused by incorrect postures during the workday were discovered, but in no case was it considered by the interviewees as a work accident or prevented them from continuing their work activity.

Regarding the psychological risks of work, work stress and satisfaction were analyzed (scale with score from 1 to 7). The average score of work satisfaction was 5.1 (*SD* = 1.4), and the average score of work stress was 4.2 (*SD* = 1.57), being stress levels higher in men (*M* = 4.73, *SD* = 1.53) as compared to women (*M* = 3.85, *SD* = 1.51). Since men usually emigrate first, they reported that the success or failure of their businesses were a family responsibility, and this burden seemed to increase stress. Problems due to stress and many hours of work without rest reverberate in finding resources and solutions to their health problems: (I-104, woman, 26 years) “Because of they work hard, they do not pay attention to their health; When any symptom of discomfort appears, they prefer to hold on and continue working (...). When they cannot stand it, they go to the doctor and take a sick leave”. Work also influences the support that Chinese immigrants can provide to other family members with health problems. In three occasions, the main researcher has observed that workers carried out telephone translations between the doctor and their families, since they could not leave their job to accompany people with less knowledge of the Spanish language to the doctor.

The distress is detected even in early ages. The testimonies of young people stand out and teenagers and second generation children are observed in their families’ businesses. Some of them work with a contract and others simply helping for a duty to respect the hierarchy and family welfare. However, they declare their dissatisfaction. On the one hand, some of them have a job not corresponding to their academic (university) degree. On the other hand, due to these working conditions, they have no leisure time: (I-56, woman, 22 years) “Parents are usually very intransigent and want their children to stay with them by their side. They think it’s good for us but it’s not like that”. In short, they do not share the same job aspiration as their parents: (I-114 woman, 19 years) “I had to work and help my parents in the store and I know what it feels like [referred in negative terms]”.

## 4. Discussion

The wide majority of People’s Republic of China (P.R.C.) immigrants come from mountainous rural areas in Southern Zhejiang; something that goes back a long way in Europe [19] and that currently is evident in countries such as Spain, Portugal, or Italy [20]. The business success of the first generation of Chinese immigrants allowed the creation of an ethnic opportunity structure that is currently consolidated. This economic success is related to the concept and attitudes towards work, as well as based on ingrained principles of Chinese culture such as self-discipline, responsibility for saving, and dedication to help the extended family (family, community, or company) and respect for authority [21]. This explains the labor and social progress of Chinese immigrants, due to the help offered by their relatives and friends both in the country of origin and destination.

In this work, the significance of having lived for longer in Spain, having more work experience, and being a self-employed worker coincides with the expectations of the migratory project: to start as an employee to finally run the business yourself, becoming a boss (laoban). In any case, the employment of Chinese immigrants practically starts from the beginning of their arrival. However, this is not shared by other immigrant groups in Spain, since if they work they do so as employees [22]. The Chinese co-ethnic network with a shared migration project facilitates the flow of information on employment opportunities and financing methods, which explains the increase since 2013 of Chinese convenience stores in Spain [23]. In addition, most of these companies do not require a large investment in technology or capital, or especially skilled labor and, therefore, some barriers can easily be overcome, maximizing profits through self-exploitation: work more hours than usual, offering personalized services, and reduced prices [21]. They hold aspirations for a better life for both themselves and their children. This desire is the motivation for family obligations and hard work [24].

Regarding the sectors occupied by the population of Chinese origin, several authors point out that restaurants and food facilities, and commerce are the most frequent activities in which they work [22,25,26,27]. This is different in the general foreign population in Andalusia, who work mainly in agriculture [28]. Although the economic crisis affected a certain foreign population in the construction sector (mainly men), this did not happen in the Chinese population, since it has never been linked to this sector [29]. This community also does not occupy the care sector (0%), something that sometimes happens among other immigrants, especially in women [30].

This study shows that the eating establishments have saturated the national market and the Chinese population has been searching for new areas of economic development [31], such as retailer (hairdressers and aesthetics, telephone stores, bazaars, fruit shops), wholesale, and imports (intense activity of warehouses concentrated in industrial estates). Finding financial success in niche markets as small business owners, the second generation in Spain has substantially lower educational ambitions and attainment than youths from other nationalities [32]. Other areas of great development in recent times are multinationals, and entrepreneurs are arriving in Spain from Huawei, Lenovo, or Dalian Wanda Group; companies with exponential growth, generating employment and wealth [33].

According to the Report on the foreign active population in Andalusia (Southern Spain), in the third quarter of 2018, the percentage of active foreigners fell by 4.73% compared to 2017 [34]. This growth in unemployment rates in the foreign population marks the seasonality of jobs, revealing an unfavorable trend for foreigners as compared to the native population. However, among Chinese men and women, the unemployment rate is practically non-existent in comparison with other groups [35,36]. Chinese immigrants consider the family as a ‘company’ whose maintenance depends on paid work and, therefore, Chinese women have not suffered unequal access to it as occurred in other immigrant groups. They are practically incorporated into the labor market in Spain since they arrived [36], adopting a main role or on equal terms with their husbands [37]. Therefore, the business activity is shown as one of the best formulas to be socially recognized, although it is more related to the obligation to contribute to family productivity than with individual aspirations. Despite this easy incorporation into the labor market, it is important to note that women play important roles in driving the business as well as the family. In the field work, it was possible to observe this aspect, in a sense that women were caring for their children while attending costumers. This has been already identified by other authors and should be addressed in future studies [38].

Although the global economic crisis generated a loss of work for the immigrant population in general [30], the propensity to self-employment of the Chinese community continued [39], which currently tends to a higher percentage of autonomous women compared to the men [6]. The positive consequences are the creation of employment and economic investment [2]. The results of this work show that more than 95% of men and women have a paid job (more than 50% as self-employed workers) and only six people reported that they have never worked.

Other differences between the Chinese community and other groups are evident in working conditions. Some studies coincide in the extent of hours dedicated to work activity, which are more than 10 hours a day in most cases [26,36,40]. In contrast to a minimum of 50 weekly hours worked by the Chinese population, the last National Survey on Working Conditions in Spain shows that the average duration of the work is 36.9 h/week [41].

In the sample of this study, less than 10% of participants work in part-time jobs, with an average of 6–7 workdays a week compared to 5–6 days of weekly work of the general Spanish population. In this respect, for the Spanish population, working at atypical times (Saturdays or Sundays, with days of more than ten hours, in shifts or at night) affects 54% working on Saturdays, 33% on Sundays, 27% have a daily shift of more than 10 h, and 22% have a night shift. Shift work affects 23% of Spanish workers [41]. While the Spanish population rests an average of 3 or 4 h in a working day, the Chinese interviewed talk about a shift work (70.9%) with more intensity, indicating that they sometimes take quick turns of a few minutes to have lunch, since they work tirelessly.

Although work is synonymous with health, ambivalence also occurs: on one hand, this is a source of wellbeing and, on the other hand, an absorbing element that does not allow people to worry about their health and even deteriorates it [42]. Occupational risk exposure is estimated to be similar with Spanish population [43]. They are mostly ergonomic risks and repetitive movements of hands or arms, forced postures, and manual handling of loads are observed during business visits [41]. The excessive working together with significant physical burdens among Chinese immigrants in this study explain why musculoskeletal problems (back pain, arthritis) are the most common problem in this group (31.6%). The musculoskeletal disorders exceed the figures collected by the European Health Survey in Spain for lumbar and cervical pain in the immigrant population (23%) [43,44].

Regarding psychosocial risks, such as overload and work rhythm, especially Chinese men show a moderate level of stress associated with work. This coincides with 35% of the Spanish employed state, that report that they work always or almost always at high speed, meeting very tight deadlines. 

The influence of the work environment is key to health, since it has been observed during fieldwork how life is made in the workplaces: provision of cribs/baby parks, having meals, reception of children after school, and help from them in business. In this respect, European workers highlight the balance between work and family life as very positive, with the monitoring of flexible time agreements when necessary [45], something that is rare among Chinese immigrants from this study, affecting family life.

Cacciani, Baglio, and Rossi (2006) indicate that the rates both work accidents and disabilities generated by them are higher in immigrant workers than in native ones [46]. However, only 15 Chinese immigrants reported an accident in the last 12 months. According to the registries of the population affiliated to the Social Security, these problems mainly take place in the construction or agrarian sectors [47] and mortality associated with accidents would be more frequent in the Spanish male population [48]. Therefore, the Chinese population would be more protected because this is not their predominant labor activity.

In addition, the reported accidents were small injuries that mostly did not require specialized health care or hospitalization. Although health problems such as colds and flu and others were more frequent than accidents, most of the workers affirm in the last 12 months that, they have worked sick. This situation is common among economic immigrants because, in view of the need to maintain their work, they are reluctant to manifest fragility and seek for attention in health systems [37].

This study has some limitations. First, a convenience sample was used, making it difficult to infer that our sample was representative of the Chinese community in Andalusia or in Spain. The high percentage of married people observed in our study was possibly a result of cultural issues among Chinese immigrants in which the value of the family predominates and also by the fact that Chinese migration is not characterized as an individual migration, but a nuclear or family migration. Nevertheless, the results are consistent with official data and other studies. Second, language barriers could be at the root of some communication difficulties and some refusals to participate, despite the fact that, in this study, three different languages were used for the same questionnaire in order to be more inclusive.

Although Chinese immigrants are young middle-aged, future studies should extend the age of the participants and incorporate ethnic Chinese (Chinese of second generation) to detect differences due to the acculturation.

## 5. Conclusions

Our study has furthered the understanding of a particular group seldom addressed in previous studies. In this context, Chinese immigrants are commonly overlooked. This is more perceived while investigating other countries outside the United States. Since this is a group rapidly whose presence is rapidly increasing in the European continent, it is important that European governments pay attention for Chinese immigrants’ needs and requests. In this matter, more studies are definitely warranted in order to provide solid information.

Our findings indicate that the Chinese immigrant population in Southern Spain has particular characteristics that should be considered. As compared to other immigrants, Chinese individuals have high rates of employability both for women and men and usually are incorporated into the services sector, upon their arrival as employees, until they establish themselves as self-employed, opening their own businesses. In relation to the impact of working conditions on their health, the Chinese immigrant population has high rates of problems related to ergonomics (i.e., musculoskeletal disorders) and stress. At the social level, the difficulties for family conciliation stand out and, therefore, they experience changes in family life routine and most participants plan to return to their countries when they attain a good economic condition. All these findings highlight the challenges faced by immigrants while searching for appropriate working conditions outside of their home country and should be considered by public health managers.

Our results could help clinicians and managers to understand Chinese immigration in European countries, to recognize health conditions related to their working conditions and to develop preventive strategies to minimize these potential problems. Helping this population in their needs such as stimulating healthy habits, minimizing social stress, increasing their participation in the Spanish society, facilitating access to documents and guaranteeing appropriate working conditions could minimize Chinese immigrants’ problems and enhance their workforce capability.

Future studies are needed in order to further investigate the role of gender in the working conditions, the influence of working hours in the health of these immigrants and how this influence is similar or different from the native population. Likewise, investigating the cross-cultural differences of Chinese immigrants among countries is another potential way to understand the role of acculturation for this group of immigrants.

In conclusion, the most common migratory reason for Chinese immigrants in Spain was to improve their economic condition. However, in the future, immigrants wish to return to their country. In order to achieve their objectives, they dedicate as much time as possible to work, even if the working hours could impact their health and quality of life. These findings are important for planning government actions in the European Union.

## Figures and Tables

**Table 1:** Sample characteristics

Variables	Male	Female	Total	Statistics
	M (SD)	M (SD)	M (SD)	*p*-Value
Age (years)	33.1 (7.2)	29.2 (7.4)	30.7 (7.6)	U = 1457.5
				*p* = *0.003*
Years residing in Spain	12.7 (5.7)	10.4 (5.5)	11.3 (5.7)	U = 4774.5
				*p* = *0.017*
	*n* (%)	*n* (%)	*n* (%)	
Sex	51 (38.3)	82 (61.7)	133 (100)	-
Marital status				
Single	11 (21.5)	34 (41.5)	45 (33.8)	χ^2^ = 7.35
Married	36 (70.6)	46 (56.1)	82 (61.7)	*p* = *0.062*
Living with a partner (not married)	3 (5.9)	2 (2.4)	5 (3.8)	
Divorced	1 (2.0)	0 (0.0)	1 (0.8)	
Level of education				
Secondary or lower	41 (80.4)	62 (75.6)	103 (77.4)	χ^2^ = 0.41
Vocational or university	10 (19.6)	20 (24.4)	30 (22.6)	*p* = *0.521*
Employment status				
Employed	50 (98.0)	78 (95.1)	128 (96.2)	χ^2^ = 0.74
Unemployed	1 (2.0)	4 (4.9)	5 (3.8)	*p = 0.649*

**Table 2:** Working conditions and health problems in Chinese immigrants.

Variable	N(%)/M(SD)
Current profession	
Bazaars and food stores	46 (36.8)
Clothing stores	23 (18.4)
Wholesale stores	15 (12)
Eating establishments	9 (7.2)
Professor	4 (3.2)
Other *	28 (22.4)
Previous laboral sector	
Commerce area	60 (56.6)
Eating establishments	30 (28.3)
Other *	16 (15.1)
Current laboral sector	
Commerce area	96 (85)
Eating establishments	9 (8)
Other *	8 (7)
How long have you been in your last job?	51.9 (42.2)
Type of work day	
≥10-Hour work day (split work day)	83 (71)
≥10-Hour work day (without rest)	13 (11.1)
Irregular work day	11 (9.4)
8-Hour work day	6 (5.1)
Part-time work day	4 (3.4)
Level of job stress	4.21 (1.57)
Level of job satisfaction	5.1 (1.38)
Accidents in the last year	
Yes	15 (11.3)
No	118 (88.7)
Accident damage	
Contusions, bruises, sprains	8 (53.3)
Deep fractures or wounds	1 (6.7)
Poisoning or intoxication	1 (6.7)
No damage	3 (20)
Perceived health problems	
Chronic back pain (cervical)	23 (17.3)
Bone pain	22 (16.7)
Gastric and intestinal problems	20 (15.2)
Headaches	16 (12.1)
Chronic back pain (lumbar)	15 (11.3)
Fever	11 (8.3)
Nervousness, depression, difficulty sleeping	10 (7.6)
Dental problems	10 (7.6)
Skin problems	10 (7.5)
Migraine or frequent headaches	8 (6.1)
Hypertension arterial and others heart’s illness	6 (4.6)
Thyroid problems	5 (3.8)
High cholesterol	4 (3.1)
Arthrosis, arthritis or rheumatism	4 (3)
Constipation	4 (3)
Liver dysfunction	2 (1.5)
Otitis	2 (1.5)
Urinary problems	2 (1.5)
Varicose veins in the legs	1 (0.8)
Asthma	1 (0.8)

* Manicure and pedicure specialist, laundry, manufacturing, delivery man, translator, teacher, and entrepreneurs who do not specify the sector.

## Supplementary Materials

The following are available online at https://www.mdpi.com/1660-4601/17/19/7063/s1, Table S1: Description of themes and categories, and distribution of verbatim quotations; Table S2: Consolidated criteria for reporting qualitative studies (COREQ): 32-item checklist.
