# Supplementary material for: “Work Like a Chinese”: Aspirations, Patterns of Work, and Working Conditions of the Chinese Immigrant Community in Southern Spain
Source: Int J Environ Res Public Health. 2020 Sep 27;17(19):7063. doi: 10.3390/ijerph17197063 (PMC7579119; doi:10.3390/ijerph17197063)
Supplement: Supplementary file 1 [file ijerph-17-07063-s001.pdf]

International Journal of
Environmental Research and Public Health

MDPI

Supplementary Materials:

Table S1. Description of themes and categories, and distribution of verbatim quotations

Themes	Description	Categories	Verbatim*
Economic improvement as a migratory reason	Narratives around the migration process: why you decide to migrate and resources or support necessary to achieve the migration objective.	Economic migration	(I-115, man, 40 years) “In Chinese culture when you are young, what you have to do is make money and then create a family and when you are older, you will retire and enjoy life more”<br><br>(I-86, man, 36 years) “I came to Spain to work more than 12 years ago. Then my wife and daughter came, but I still have a child in China with my mother. I cannot bring it until I can cope economically with the maintenance of another person”
		Economic support network	(I-14, woman, 21 years) “The person who arrives is always with a link; let's say a tie (…). They house you [other Chinese immigrants] in their house, they give you work”<br><br>(I-55, woman, 33 years) “You come and work in the store of your brother, your uncle, cousin ... They offer you a work contract, and, when you have their conditions, you set up your work”<br><br>(I-132, woman, 41 years) “To come to Spain, my parents were helped by my uncles with funding. And then, my uncles decided to come and my parents did the same when they were already in Spain ”

Conception of Work	Positive or negative perception of work, aspects of Chinese work culture and differences with the Spanish one.	Conception of work	(I-114, woman, 19 years) "The 2nd generation or university people are changing. But not the usual ones that comes here, who just work, work and work"<br><br>(I-107, woman, 33 years) "Especially the first generation of Chinese immigrants, they do not know Spanish, do not know anyone, have no Spanish friends; they can not integrate. You can not do anything but be in a store"<br><br>(I-5, man, 35 years) "I've been working in Spain for 10-11 years. Although I am right now unemployed, I help my wife in the store, but I do not charge unemployment. The Chinese in general think that if it is possible there is no need to bother anyone or the government [reference to receiving unemployment benefits]".<br><br>(I-73, man, 34 years) "Holidays are pejorative (...). There are things that Chinese will never understand. The issue of dismissals (...), we do not understand what to pay for vacations"
Labor Sector	Career trajectory of the interviewees in the different labor sectors and professional activities.	Labor Sector	(I-50, man, 43 years) "Here until now were the restaurants (...). There are many people who are now engaged in telephony (...) greengrocers and shops 'all at one euro'. But there are also many people who are importers. That is another different scale (...) they are earning a lot of money. Most are young people, 30-40 years old"<br><br>(I-122, woman, 23 years) "They work in the restaurants, bazaars, now in industrial parks. Some of them are working in banks and similar, but that is the people of my generation with higher level of education"
Work Conditions	Hiring, current and expected work regime, type of working day and working hours.	Work regime	(C-100, woman, 25 years) "In the first years we worked hard and on behalf of others with the idea of being able to start our own business. That is the goal of every Chinese citizen who comes to Spain and I believe that to any other country, I think we are very enterprising. The Chinese seek

			in the shortest possible period of time, to have savings to start their own business with the financial support of their most direct family circle”<br><br>(C-85, man, 37 years) “All Chinese immigrants have done the same. The first example was that of this man [He mentions the name of the owner of a food store specializing in Chinese products]. He arrived here in the year 86 and first worked with his uncle in a restaurant and then opened his business, one of the first Chinese stores in the city of Seville”
		Workday	(I-93, woman, 34 years) “I believe that the workday is longer than that of any worker, for the purpose of earning the maximum amount of money to be able to establish on our own”<br><br>(I-23, men, 23 years) “Chinese work every day from Monday to Monday and from 10 a.m. to 10 p.m. Chinese do not mind working 10-12-15 hours if it is necessary. Those who work for others temporarily do so until they can work on their own. I do not think they have a good quality of life with respect to work”<br><br>(I-7, man, 18 years) “My parents work 12 hours a day, from Monday to Saturday. Sometimes they use Sundays to rest, but most of them go to the stores to look for merchandise, that is, they do not have any time to rest. That of the stereotype that Chinese works hard is true”
Occupational health	Health problems caused at work or because of it. Physical and psychological aspects related to working conditions. Perception and incidence of accidents at work	Physical and psychosocial problems	(I-39, man, 32 years) “People who work in a store have back and muscle problems, because of being loading and unloading things on the shelves or sitting all day (...). I think that being 12 or 14 hours in a store day after day affects us negatively”<br><br>(I-10, man, 32 years) “Sedentary first, then the bad diet habits... everything produced by this type of work. At least the one who works in a store and spends so many hours sat down”<br><br>(I-66, woman, 37 years) “Work conditions make you “to cannot take care of yourself. Many times you get tired of work at night so you eat anything”

	(I-15, man, 35 years) "The risks are related to work in industrial ships and cargo ships, and the contact with machinery, elevator … as any other person who work in this places"<br><br>(I-54, woman, 33 years) "Because of the impossibility of leaving work for a few hours, they don't go very frequently to the health care center and this, at the end, can affect them, since they are not diagnosed and treated appropriately"<br><br>(I-63, woman, 36 years) Due to stress and so many hours of work without rest, I believe that work affects. I usually rest one day a week but ... I consider it still very intense.<br><br>(I-89, man, 42 years) Although I work hard to support my family, sometimes I feel lonely. I receive invitations from friends to go out but I can't for the job.
Work accidents	(I-109, man, 40 years) A few days ago I noticed a tug while working in the warehouse. I have a little pain but I prefer to endure or take some herbs, because I cannot lose half a day of work in going to the doctor.<br><br>(I-84, woman, 32 years) I have not had accidents. Only the other day I doubled my foot down a ladder. The doctor recommended resting at home, but I have to keep working.

* This table shows the most complete verbatim of the results. The ideas reflected in all of them are shared in all cases by more than 80% of the interviewees. No interviewee shows a radically opposite point of view

Table S2. Consolidated criteria for reporting qualitative studies (COREQ): 32-item checklist

No	Item	Guide questions/description	Response
Domain 1: Research team and reflexivity			
Personal Characteristics			
1.	Interviewer/facilitator	Which author/s conducted the interview or focus group?	All the interviews were conducted by the main author, BB.
2.	Credentials	What were the researcher's credentials? E.g. PhD, MD	BB, SBT, RD, EFG, MGC and GL were PhD. RJFC was PhD student.
3.	Occupation	What was their occupation at the time of the study?	Researcher's occupations at the time of the study: research professor.
4.	Gender	Was the researcher male or female?	BB, EFG, MGC and RD were females. SBT, RJFC and GL were males.
5.	Experience and training	What experience or training did the researcher have?	All researchers had experience in carrying out qualitative research. BB has been trained to conduct interviews and RD has training in social research.
Relationship with participants			
6.	Relationship established	Was a relationship established prior to study commencement?	No, there wasn't.
7.	Participant knowledge of the interviewer	What did the participants know about the researcher? e.g. personal goals, reasons for doing the research	Name, occupation, reasons for doing the research.
8.	Interviewer characteristic s	What characteristics were reported about the interviewer/facilitator? e.g. Bias, assumptions, reasons and interests in the research topic	Name, occupation, contact method , reasons for doing the research.
Domain 2: Study design			
Theoretical framework			

9.	Methodological orientation and Theory	What methodological orientation was stated to underpin the study? e.g. grounded theory, discourse analysis, ethnography, phenomenology, content analysis	Phenomenological and ethnographic approach with a discourse and content analysis.
Participant selection			
10.	Sampling	How were participants selected? e.g. purposive, convenience, consecutive, snowball	Convenience sampling and snowball sampling.
11.	Method of approach	How were participants approached? e.g. face-to-face, telephone, mail, email	Face to face.
12.	Sample size	How many participants were in the study?	133 Chinese immigrants
13.	Non-participation	How many people refused to participate or dropped out? Reasons?	279 for laboral reasons (mainly lack of time).
Setting			
14.	Setting of data collection	Where was the data collected? e.g. home, clinic, workplace	Mainly in workplace, and other quiet and comfortable place chosen by the participant.
15.	Presence of non-participants	Was anyone else present besides the participants and researchers?	In all businesses there were other workers or family members (children among them).
16.	Description of sample	What are the important characteristics of the sample? e.g. demographic data, date	Chinese workers in their 30's emigrated to Spain.
Data collection			
17.	Interview guide	Were questions, prompts, guides provided by the authors? Was it pilot tested?	Yes, they were. / Yes, it was.
18.	Repeat interviews	Were repeat inter views carried out? If yes, how many?	No, they weren't.
19.	Audio/visual recording	Did the research use audio or visual recording to collect the data?	Audio recording.
20.	Field notes	Were field notes made during and/or after the interview or focus group?	Yes, they were (field notes).
21.	Duration	What was the duration of the inter views or focus group?	Average 30-50 minutes
22.	Data saturation	Was data saturation discussed?	Yes, it was.

International Journal of
Environmental Research and Public Health

MDPI

23.	Transcripts returned	Were transcripts returned to participants for comment and/or correction?	Reviewed by 2 participants.
		Doman 3: Analysis and findings	
Data analysis			
24.	Number of data coders	How many data coders coded the data?	Three (BB, RDC and SB).
25.	Description of the coding tree	Did authors provide a description of the coding tree?	Yes, we did.
26.	Derivation of themes	Were themes identified in advance or derived from the data?	Themes were derived using both methods.
27.	Software	What software, if applicable, was used to manage the data?	NUDIST Nvivo 11.
28.	Participant checking	Did participants provide feedback on the findings?	Reviewed by 2 participants.
Reporting			
29.	Quotations presented	Were participant quotations presented to illustrate the themes/findings? Was each quotation identified? e.g. participant number	Yes, there were. / Yes, there was.
30.	Data and findings consistent	Was there consistency between the data presented and the findings?	Yes, there was.
31.	Clarity of major themes	Were major themes clearly presented in the findings?	Yes, they were.
32.	Clarity of minor themes	Is there a description of diverse cases or discussion of minor themes?	Yes, there is.

Developed from: Tong, A. Sainsbury, P., and Craig, J. 2007. Consolidated criteria for reporting qualitative

research (COREQ): A 32- ítem checklist for interviews and focus group. Int. J. Qual. Health Care 19: 349-357.
